# Interval Sentinel Lymph Nodes: An Unusual Localization in Patients with Cutaneous Melanoma

**DOI:** 10.1155/2011/506790

**Published:** 2011-05-04

**Authors:** A. M. Manganoni, R. Farfaglia, E. Sereni, C. Farisoglio, C. Pizzocaro, D. Marocolo, F. Gavazzoni, L. Pavoni, P. Calzavara-Pinton

**Affiliations:** ^1^Department of Dermatology, University Hospital Spedali Civili, 25123 Brescia, Italy; ^2^Department of II Surgery, University Hospital Spedali Civili, 25123 Brescia, Italy; ^3^Department of Nuclear Medicine, University Hospital Spedali Civili, 25123 Brescia, Italy; ^4^Department of I Pathology, University Hospital Spedali Civili, 25123 Brescia, Italy

## Abstract

*Background*. Recent studies have demonstrated that there exists a great variation in the lymphatic drainage in patients with malignant melanoma. Some patients have drainage to lymph nodes outside of conventional nodal basins. The lymph nodes that exist between a primary melanoma and its regional nodal basin are defined “interval nodes”. Interval node occurs in a small minority of patients with forearm melanoma. We report our experience of the Melanoma Unit of University Hospital Spedali Civili Brescia, Italy. *Methods*. Lymphatic mapping using cutaneous lymphoscintigraphy (LS) has become a standard preoperative diagnostic procedure to locate the sentinel lymph nodes (SLNs) in cutaneous melanoma. We used LS to identify sentinel lymph nodes biopsy (SLNB) in 480 patients. *Results*. From over 2100 patients affected by cutaneous melanoma, we identified 2 interval nodes in 480 patients with SLNB . The melanomas were both located in the left forearm. The interval nodes were also both located in the left arm. *Conclusion*. The combination of preoperative LS and intraoperative hand-held gamma detecting probe plays a remarkable role in identifying these uncommon lymph node locations. Knowledge of the unusual drainage patterns will help to ensure the accuracy and the completeness of sentinel nodes identification.

## 1. Introduction

Recent studies have demonstrated that there exists a great variation in the lymphatic drainage among patients with malignant melanoma [1–11]. While most melanomas show lymphatic drainage to usual nodal basins (axillary, inguinal, and cervical regions), some patients also have drainage to lymph nodes outside these regions [5, 6, 9, 12]. Lymphatic nodes in the area between the primary melanoma and the regional basins are called “in-transit nodes”, “interval nodes”, or “interval sentinel lymph nodes”, and by definition are also SLNs [2–5, 9, 10].

We describe our experience at the Melanoma Unit of University Hospital Spedali Civili Brescia, Italy. 

## 2. Materials and Methods

A retrospective study was performed considering 480 patients selected from over 2100 Caucasian patients affected by cutaneous melanoma with SLNB. These patients were followed up by the Melanoma Unit of University Hospital Spedali Civili Brescia, Italy. All patients gave an informed consent to be entered into the database. They were staged with the use of American Joint Committee on Cancer (AJCC) staging classification [13–15]. The combination of preoperative LS and intraoperative hand-held gamma detecting probe allows detection of the SLNs in 99,0% of the patients with malignant melanoma [5, 6, 9, 16]. 

## 3. Results

From over 2100 patients affected by cutaneous melanoma, we identified 2 interval nodes from 480 patients with SLNB. The interval nodes were located in the left arm. All patients were Caucasian.

**Case 1**

A 26-year-old Caucasian man in good general health, presented to our clinic in July 2010 with an asymptomatic irregular hyperpigmented lesion of 5 × 5 mm dimension, situated in the left forearm. Both the anamnestic information and the clinical and dermoscopic characteristic of the lesion provided the basis for the diagnosis of melanoma. We performed an excision of the pigmented lesion, and the histological examination showed it to be a melanoma: Breslow thickness was 2.6 mm, mitoses were 2/mm (2), and ulceration was absent.

Clinical examination and preoperative total-body computed tomography (TC Total Body), showed no lymph node or visceral metastases. We performed an ultrasonography (US) at the conventional nodal basins (armpits, groin, and neck) and also to the brachial soft tissues. The ultrasound examination showed a small superficial swelling area located in the left mid-arm.

**Figure 1:**
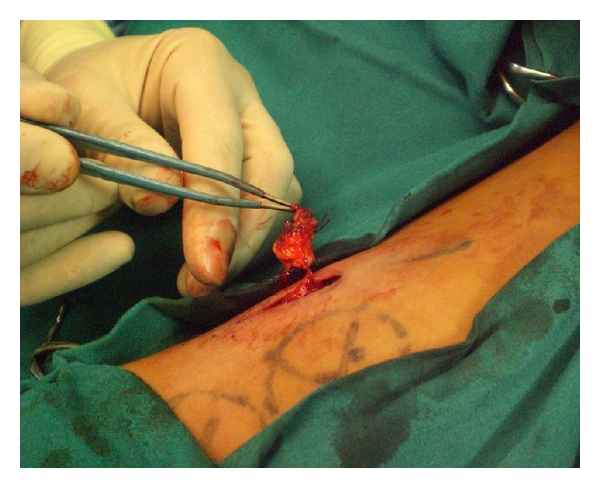
Case 1. A 26-year-old man with a left forearm melanoma. Intraoperatory image of an interval node of the mid-arm.

In conformity with the AJCC Classification [15] we performed a wide excision of the melanoma scar and an SLNB. Before the excision, SLNB was carried out with LS with three intradermic injection of the radioactive substance (22.2 Mbq of 99mTc) in the left forearm, near the scar area. Two focal areas of increased uptake were seen in the left axilla and at the left mid-arm (Figure 1). Therefore, we performed a selective surgical excision of SLNs in the left axilla and left mid-arm for histological examination [14].

Metastasis of melanoma to both mid-nodes and left axilla were identified histologically. In conformity with the AJCC Classification [14, 15], we performed a complete axillary lymphadenectomy, with a full levels (I, II, and III) dissection [14, 15]. We also performed a surgical dissection of the mid-arm superficial lymphatic tissues “en bloc” with surgical scar, the subcutaneous tissue, and the basilic vein [17]. The dissection then proceeded proximal to the medial intermuscular septum, deep to the basilic vein near the brachial neurovascular pedicle. The histological examination showed no metastasis in the left axilla (0/22) and in the left mid-arm (0/3). Thus the patient was sent to a regular followup.

**Case 2**

A 63-year-old white female in good general health came to our attention for an asymptomatic irregular hyperpigmented lesion with dimensions 1 × 0,8 cm, situated in the left forearm. The anamnestic information and the dermatoscopic characteristics indicated that it could be a thick melanoma. We performed a surgical excision, and the subsequent hystological examination showed it to be a melanoma: Breslow thickness was 2.3 mm, mitoses were 2/mm (2), and ulceration was absent.

**Figure 2:**
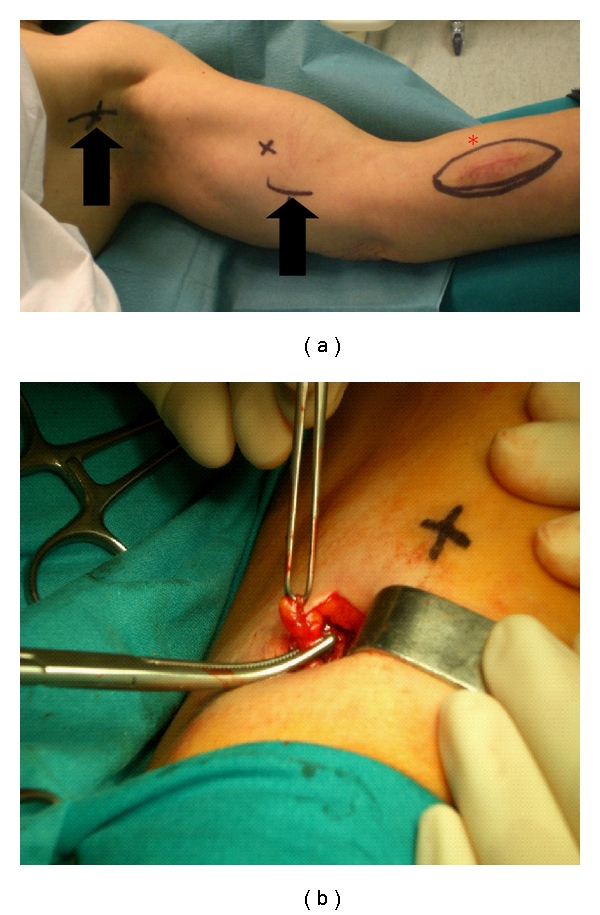
Case 2 patient. A 63-year-old woman with melanoma on the left forearm. The location of the primary tumor is indicated with a red star (*) and the areas of increased uptake with arrows (a). Intraoperatory image of interval node of the mid-arm (b).

Clinical examination, preoperative total-body computed tomography (TC Total Body), and lymphnodes ultrasonography showed no lymph node or visceral metastases. In conformity with the AJCCClassification [14], we performed a wide reexcision of the melanoma scar and an SLNB. The LS showed two focal areas of increased uptake at the left mid-arm and in the left axilla (Figures 2(a) and 2(b)). No metastases were identified by the histological examination of the sentinel nodes excised, and thus the patient was sent to a regular followup.

The nodes were not palpable on the medial side of the upper arm.

## 4. Conclusions

Recent studies have demonstrated that there exists a great variation in the lymphatic drainage between patients with malignant melanoma [1–11, 18–20]. Although most melanomas exhibit lymphatic drainage to conventional nodal basins (i.e., the cervical, axillary, and inguinal nodes), some patients show drainage to lymph nodes outside of these basins [5, 9–11, 20]. The lymph nodes that exist between a primary melanoma and its regional nodal basin are termed “interval nodes” [5, 11, 20]. The incidence of metastatic in-transit nodes was between 14% and 22% [5, 21]. Interval nodes were more common in melanoma of the trunk than in those of the lower limbs [5, 21].

Although this interval nodes positivity is uncommon, the literature has showed that in more than 85% of cases reported with a metastatic disease in the interval node, this is often the only site of nodal metastasis [5, 11].

Interval SLN can be reliably detected only by detailed preoperative LS and the intraoperative use of a handheld gamma probe. We identified two upper arm interval nodes in 480 patients submitted to SLNB for primary cutaneous melanoma at Spedali Civili di Brescia, Center Melanoma Unit.

Both the interval nodes were located in the left arm.

We would like to underline that lymph nodes can be observed in unusual localization, not only in areas with well-known drainage to regional lymph nodes. LS plays a remarkable role in identifying these uncommon lymph node locations. Knowledge of the unusual drainage patterns outlined in this paper will help to ensure the accuracy and the completeness of sentinel nodes identification.
